# lncRNA LENGA sponges miR-378 to promote myocardial fibrosis in atrial fibrillation

**DOI:** 10.1515/med-2023-0831

**Published:** 2023-11-14

**Authors:** Liting Wu, Bingjing Gao, Mengyuan Shen, Lu Wei, Zhumeng Li, Wenfang Zhuang

**Affiliations:** Medical Laboratory, Shidong Hospital Affiliated to University of Shanghai for Science and Technology, Shanghai, 200438, China; Medical Laboratory, Shidong Hospital Affiliated to University of Shanghai for Science and Technology, 999 Shiguang Road, Yangpu District Shanghai, 200438, China

**Keywords:** atrial fibrillation, myocardial fibrosis, LENGA, miR-378

## Abstract

miR-378 is known to suppress myocardial fibrosis, while its upstream regulators have not been identified. lncRNA LENGA is a recently identified lncRNA in cancer biology. We observed the altered expression of LENGA in atrial fibrillation (AF) patients and predicted its interaction with miR-378. We then explored the interaction between LENGA and miR-378 in AF. Angiotensin-II (Ang-II)-induced human atrial cardiac fibroblasts and human atrial muscle tissues were collected and the expression of LENGA and miR-378 was determined by RT-qPCR. The interaction between LENGA and miR-378 was analyzed through bioinformatics analysis and confirmed by RNA pulldown assay. Cell proliferation and collagen production were analyzed through *in vitro* assay to analyze the role of LENGA and miR-378 in MF. AF patients showed increased expression of LENGA and deceased expression of miR-378 compared to the sinus rhythm group. LENGA and miR-378 interacted with each other, while they are not closely correlated with each other. Overexpression assay showed that LENGA and miR-378 overexpression failed to affect each other’s expression. LENGA promoted collagen production and proliferation of Ang-II-induced atrial fibroblasts, while miR-378 played opposite roles. Moreover, LENGA suppressed the function of miR-378. Therefore, LENGA may sponge miR-378 to promote MF in AF.

## 1 Introduction

Atrial fibrillation (AF), also called AF or AFib, is an upper ventricular rapid arrhythmia, accompanied by uncoordinated atrial electrical activity and the resulting ineffective atrial contraction [1,2]. Without proper control and treatment, AF often causes the formation of blood clots in the heart [3]. It is estimated that AF is affecting about 1% of the population worldwide, and its incidence is predicted to be significantly increased by 2050 [4,5,6]. The crude mortality of AF is about 70.7 per 100,000 person per year [7,8]. Patients with AF are usually treated with certain therapies to reset the heart rhythm, medications to prevent and treat blood clots, and catheter procedures [9]. However, treatment outcomes in many cases are still unsatisfactory and the mortality is unacceptably high [9]. Therefore, novel therapies are still needed.

Understanding the molecular mechanism underlying the initiation and development of AF is the key for the development of AF. Patients with AF experience pathological changes in atrial myocardium, such as glycogen accumulation, mitochondrial abnormalities, loss of sarcomeres, and increased cardiomyocyte volume [10,11]. Atrial structural remodeling in AF patients is characterized by atrial fibrosis, which is caused by dysregulated atrial fibroblast proliferation and the increased extracellular matrix deposition [10,11]. Therefore, regulating the proliferation of atrial fibroblast may contribute to the recovery of AF.

Previous studies have characterized a big number of non-coding RNAs involved in the development of AF [12,13,14], and some of these RNAs are potential targets to treat AF [14,15,16]. For example, lncRNA PVT1 regulates atrial fibrosis via miR-128-3p-SP1-TGF-β1-Smad axis in AF [17]. lncRNA-LINC00472 contributes to the pathogenesis of AF by reducing the expression of JP2 and RyR2 via miR-24 [18]. Moreover, microRNAs (miRNAs) are endogenous ∼23-nt RNAs that can complementarily bind to messenger RNAs (mRNAs) of protein-coding genes and lead to their regulation post-transcriptionally [19,20]. Most published studies have focussed on their effect on the immune system or in cancer, while only a few reported their role in cardiovascular disease [21,22,23,24]. For instance, miR-378 has been reported to suppress myocardial fibrosis [25]. However, upstream regulators of miR-378 in this process are unknown. lncRNA LENGA is a recently identified lncRNA in cancer biology [26], which acts as a tumor suppressor in gastric cancer through BRD7/TP53 signaling [26]. We observed the altered expression of LENGA in AF patients and predicted its interaction with miR-378. We then explored the interaction between LENGA and miR-378 in AF.

## 2 Materials and methods

### 2.1 Patients and tissue samples

Research subjects of the present study included the (AF, *n* = 37) group and sinus rhythm group (SR, *n*  =  32). All these patients received surgeries of heart valve replacement between May 2020 and May 2022 at Shidong Hospital Affiliated to University of Shanghai for Science and Technology after Ethics Committee of this hospital approved this study. Subsequently, peripheral blood samples were collected from all subjects to measure their expression of Collagen I, Collagen III, miR-378 and LENGA. In addition, cardiac tissue samples were collected from the left atrial free wall near the interatrial septum in both AF patients (the AF group, *N* = 37) and healthy subjects (the control group, *N* = 32) to measure the expression of Collagen I, Collagen III, miR-378, and LENGA in tissue samples. All of these patients signed informed consent. Research subjects of this study excluded the ones with a history of hyperthyroidism, infective endocarditis, coronary atherosclerotic heart disease, liver and kidney dysfunction, and chronic pulmonary heart disease. During surgery, patients’ atrial muscle tissues were collected and stored in liquid nitrogen. Clinical data of SR and AF groups are presented in Table 1.

**Table 1:** Clinical data of AF and SR groups

	AF (*n* = 37)	SR (*n* = 32)
Age	52.3 ± 10.8	53.1 ± 13.1
Male gender	18	16
**NYHA classification**		
I/II	4	3
III	25	24
IV	8	5
SBP (mm Hg)	120.19 ± 11.12	115.19 ± 11.09
DBP (mm Hg)	75.82 ± 11.11	72.12 ± 8.37
LAD (cm)	4.67 ± 0.29*	3.12 ± 0.11
RAD (cm)	3.66 ± 0.31	3.50 ± 0.31
LVEF (%)	55.21 ± 2.99	57.03 ± 3.11

*P < 0.05.

### 2.2 Human atrial cardiac fibroblasts and cell culture

Human atrial cardiac fibroblasts, which were cryopreserved at passage one, were purchased from Innoprot (Biscay, Spain). Cells were cultivated in Fibroblast Medium-2 (P60108-2, Innoprot) at 37°C (95% humidity and 5% CO_2_). Cells were collected from passage three to five for further applications.

### 2.3 Cell transfection

Overexpression vector of LENGA was established with pcDNA3.1 as a backbone. Mimic of miR-378 (5′ACUGGACUUGGAGUCAGAAGGC-3′) and negative control (NC) miRNA were designed and synthesized by Sangon (Shanghai, China). Human atrial cardiac fibroblasts collected from passage three to five were used to prepare single cell suspensions, which were transfected with the vector-expressing LENGA and/or miR-378 mimic using Lipofectamine™ 2000 Transfection Reagent (Invitrogen). Transfections were conducted following manufacturer’s instruction. Cells were harvested at 48 h post-transfection to isolate total RNA samples, which were used in RT-qPCR to check the efficiency of transfections.

### 2.4 RNA isolation

Human atrial cardiac fibroblasts and atrial muscle tissues were subjected to RNA isolation using TRIzol reagent (Invitrogen). Briefly, cells were harvested and tissue samples were used to prepare tissue powder in liquid nitrogen. Then, TRIzol reagent was mixed with the harvested cells or tissue samples to a ratio of 10 to 1. Chloroform purification was performed twice to remove protein contamination. Phenol precipitation was down to collect RNA. RNA samples were analyzed using Bioanalyzer. DNase I digestion was performed to completely remove DNA contamination.

### 2.5 RT-qPCR

SSRT IV kit (Invitrogen) was used to prepare cDNA samples with total RNA samples as template. SYBR Green PCR Mix (TaKaRa) was then used to prepare qPCR mixture. qPCR was performed on CFX Opus 96 Real-Time PCR System (Bio-Rad) to determine the expression levels of Collagen I, Collagen III, LENGA and miR-378. The method of 2^−ΔΔCt^ was used to analyze Ct values to calculate the fold changes of gene expression levels.

### 2.6 BrdU assay analysis of cell proliferation

A BrdU kit (Abcam, Cambridge, MA, USA) was applied to determine the proliferation of human atrial cardiac fibroblasts. Cells were collected at 48 h post-transfection and further cultivated in fresh medium containing 1 μM Angiotensin-II (Ang-II) (Sigma-Aldrich, St. Louis, MO, USA) for 24 h. After that, cells were stained with BrdU for 1 h, followed by incubation with anti-BrdU antibody for 1 h. To quantify cell proliferation, OD values at 450 nm were measured.

### 2.7 RNA pulldown assay

RNA transcripts of LENGA and NC were prepared using HiScribe T7 *In Vitro* Transcription Kit (E2030, NEB). Biotin labeling at the 3′ end was performed using Pierce™ RNA 3′ End Biotinylation Kit. Cell lysates were prepared using human atrial cardiac fibroblasts and incubated with Biotin labeled LENGA and NC RNAs at 25°C for 1 h. Streptavidin agarose beads (Invitrogen) were then used to pulldown RNA complexes. RNA complexes were purified using TRIzol (Invitrogen), followed by RT-qPCR to determine the expression of miR-378.

### 2.8 Statistical analysis

GraphPad Prism 6 software was used to perform all statistical analyses. Data were expressed as mean ± standard deviation values of three biological replicates or average values of three technical replicates and were compared by unpaired *t* test and One-way analysis of variance, respectively. Correlations were analyzed using Pearson’s correlation coefficient. *p*  <  0.05 was statistically significant.


**Ethics approval and consent to participate**: This study was approved by Ethics Committee of the Shidong Hospital Affiliated to University of Shanghai For Science and Technology. The study followed the tenets of the Declaration of Helsinki, and informed written consent was obtained from all patients and controls after we explained the nature and possible consequences of the study.

## 3 Results

### 3.1 Analysis of Collagen I, Collagen III, LENGA, and miR-378 expression in AF and SR groups

**Figure 1:**
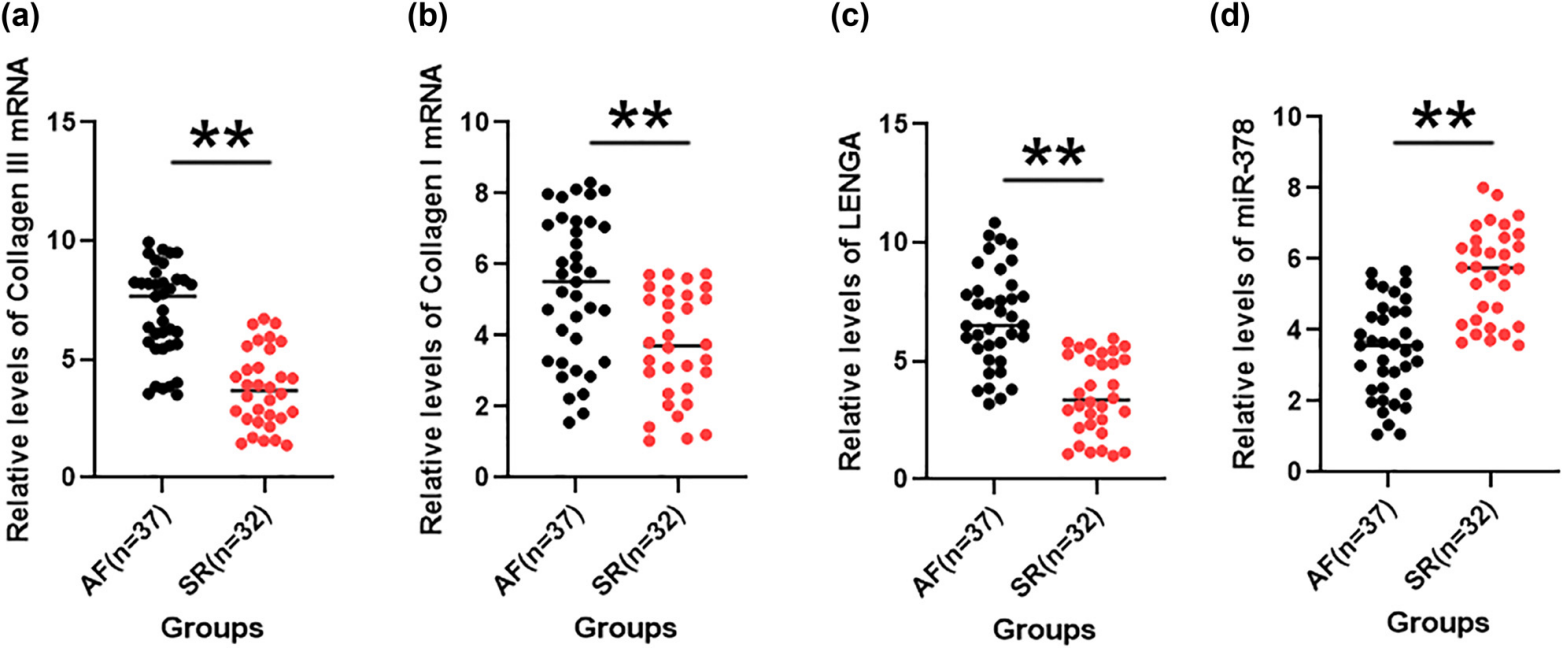
Analysis of Collagen I, Collagen III, LENGA, and miR-378 expression in AF and SR groups. Expression of Collagen I (a) and Collagen III (b) mRNA, as well as LENGA (c) and miR-378 (d), was determined through RT-qPCR in both AF and SR groups. Data were expressed as average values of three qPCR replicates. **p < 0.01.

Expression of Collagen I and Collagen III mRNA, as well as LENGA and miR-378 was determined through RT-qPCR in both AF and SR groups. Compared to SR group. The AF group showed significantly increased expression of both Collagen I (Figure 1a, *p* < 0.01) and Collagen III (Figure 1b, *p* < 0.01) mRNAs. Moreover, AF patients showed increased expression of LENGA (Figure 1c, *p* < 0.01) and deceased expression of miR-378 (Figure 1d, *p* < 0.01) compared to the SR group. Therefore, altered expression of Collagen I, Collagen III, LENGA, and miR-378 are likely participate in AF.

### 3.2 LENGA showed a negative correlation to Collagen I and Collagen III mRNA in AF samples, but not in SR samples

**Figure 2:**
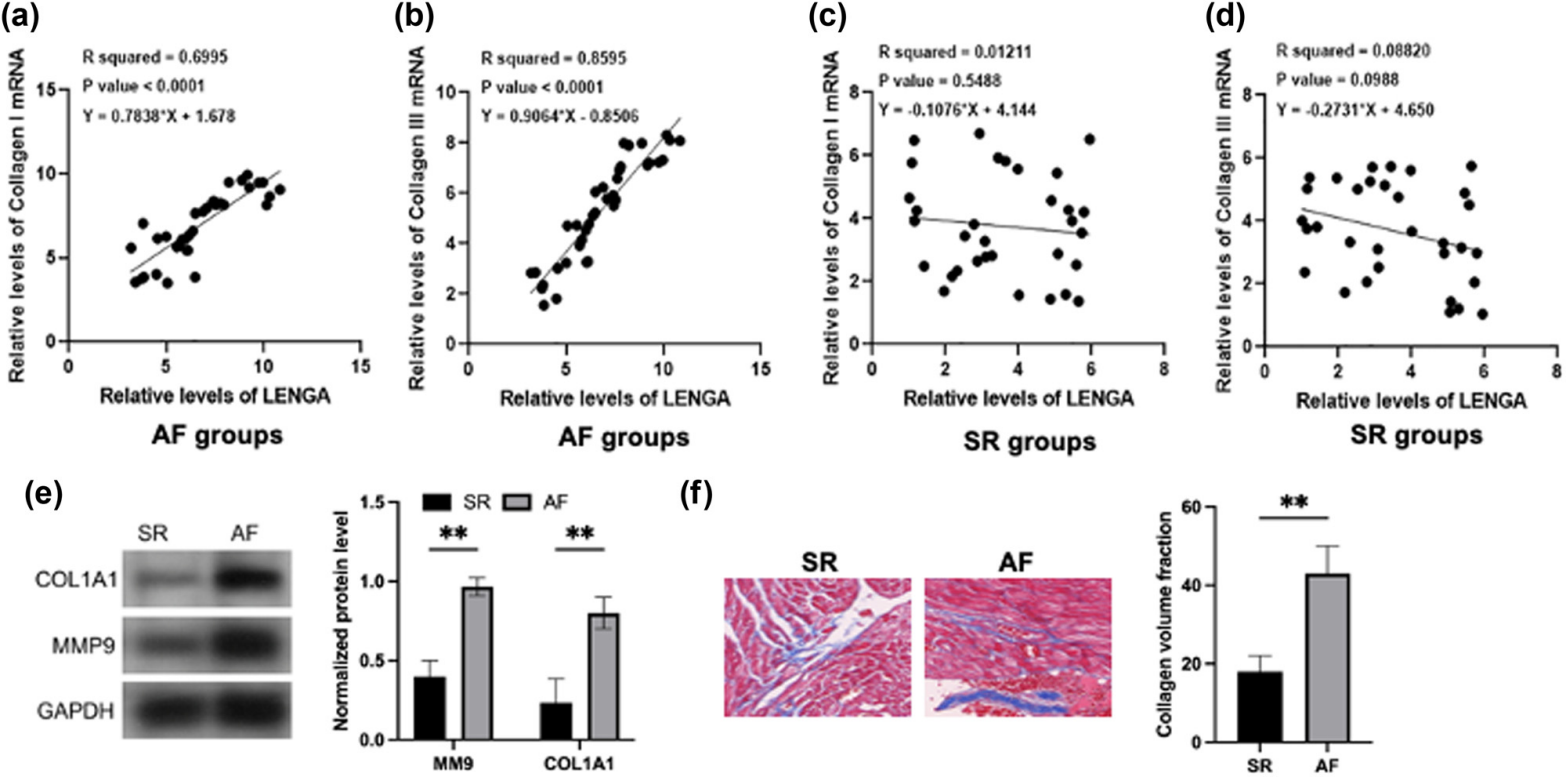
LENGA showed a negative correlation to Collagen I and Collagen III mRNA in AF samples, but not in SR samples. The correlations of LENGA with Collagen I (a) and Collagen III (b) mRNA across AF samples were analyzed by Pearson’s correlation coefficient. The same method was applied to analyze the correlations of LENGA with Collagen I (c) and Collagen III (d) mRNA across SR samples. Western blot analysis showed protein expression of MMP9 and COL1A1 in the AF and SR groups (e). Histopathological changes and the collagen volume fraction in left atrium after staining with Masson’s trichrome stain in the AF and SR groups (f). **p < 0.01.

The correlations of LENGA to Collagen I and Collagen III mRNA across AF samples and SR samples were analyzed by Pearson’s correlation coefficient. Across AF samples, LENGA was positively and significantly correlated with both Collagen I mRNA (Figure 2a) and Collagen III mRNA (Figure 2b). Across SR samples, LENGA showed no close correlation to Collagen I (Figure 2c) and Collagen III mRNA (Figure 2d). And we also detected that COL1A1 and MM9 (they are fibrotic marker) expression level was increased in the AF groups comparing to the SR groups (Figure 2e). In addition, the collagen volume fraction is more augmented in the AF groups than in the SR groups (Figure 2f). Therefore, LENGA may affect Collagen I and Collagen III in AF to affect disease progression.

### 3.3 LENGA and miR-378 interacted with each other, but showed no role in each other’s expression

**Figure 3:**
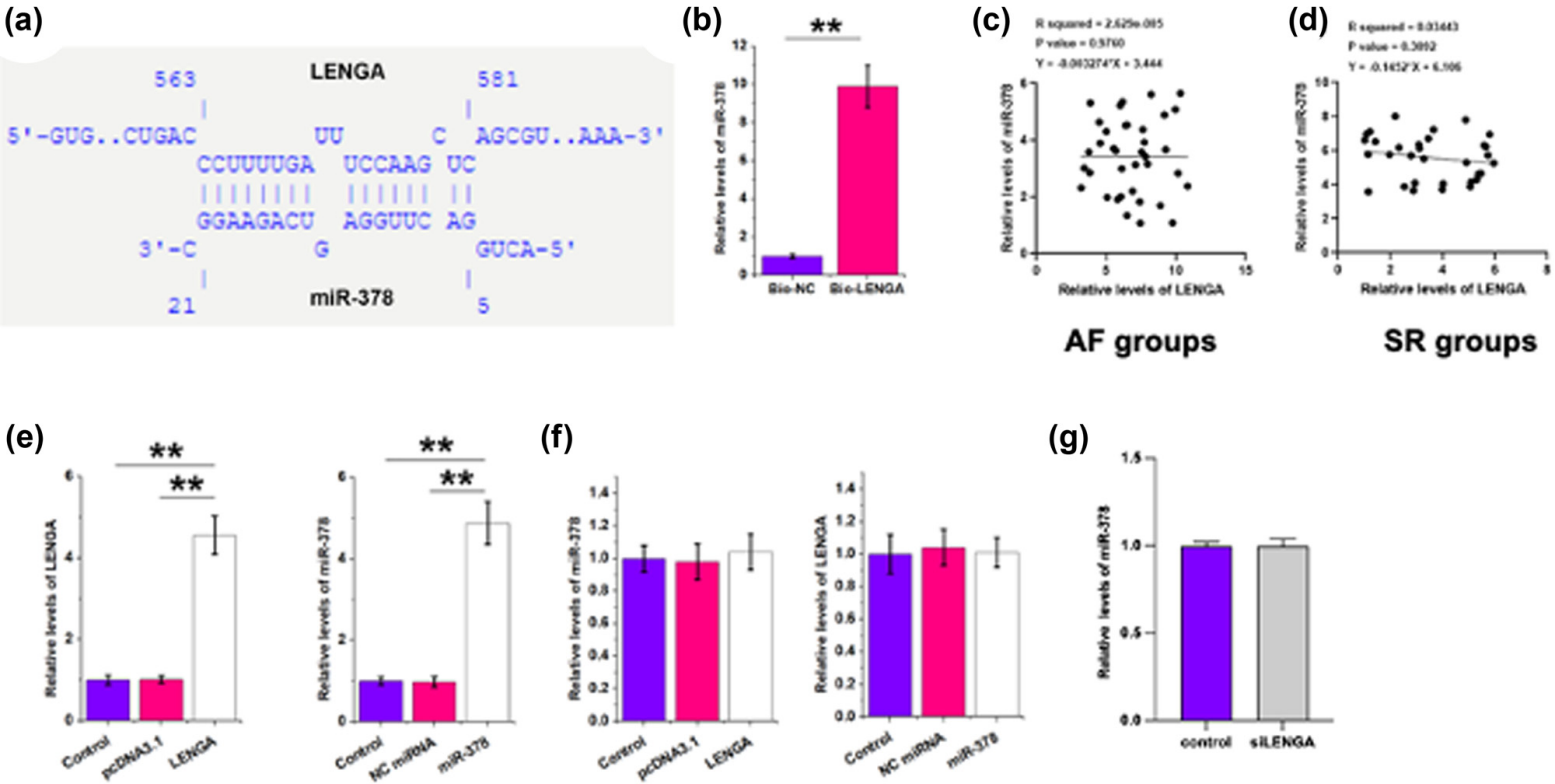
LENGA and miR-378 interacted with each other but showed no role in each other’s expression. IntaRNA 2.0 was used to predict the interaction between LENGA and miR-378 (a), which was confirmed by RNA pulldown assay (b). The correlations between LENGA and miR-378 across AF (c) and SR (d) samples were analyzed by Pearson’s correlation coefficient. LENGA and miR-378 were overexpressed in human atrial cardiac fibroblasts (e), and the role of LENGA and miR-378 in each other’s expression was also analyzed by RT-qPCR (f). Knocked down LENGA in human atrial cardiac fibroblasts first, then check the expression of miR-378 (g). **p < 0.01.

Based on intaRNA 2.0 prediction, the sequences of LENGA and miR-378 may form multiple base pairs (Figure 3a). RNA pulldown assay was performed to verify this interaction. Compared to the Bio-NC pulldown group, the Bio-LENGA pulldown group showed significantly increased levels of miR-378 RNA accumulation, further confirming the interaction between them (Figure 3b, *p* < 0.01). The correlations between LENGA and miR-378 across AF (Figure 3c) and SR (Figure 3d) samples were analyzed by Pearson’s correlation coefficient. Interestingly, they are not closely correlated with each other across these samples. LENGA and miR-378 were overexpressed in human atrial cardiac fibroblasts (Figure 3e). Overexpression and knock down assay showed that LENGA and miR-378 overexpression failed to affect each other’s expression (Figure 3f and g).

### 3.4 The role of LENGA in collagen production and cell proliferation was affected by miR-378

**Figure 4:**
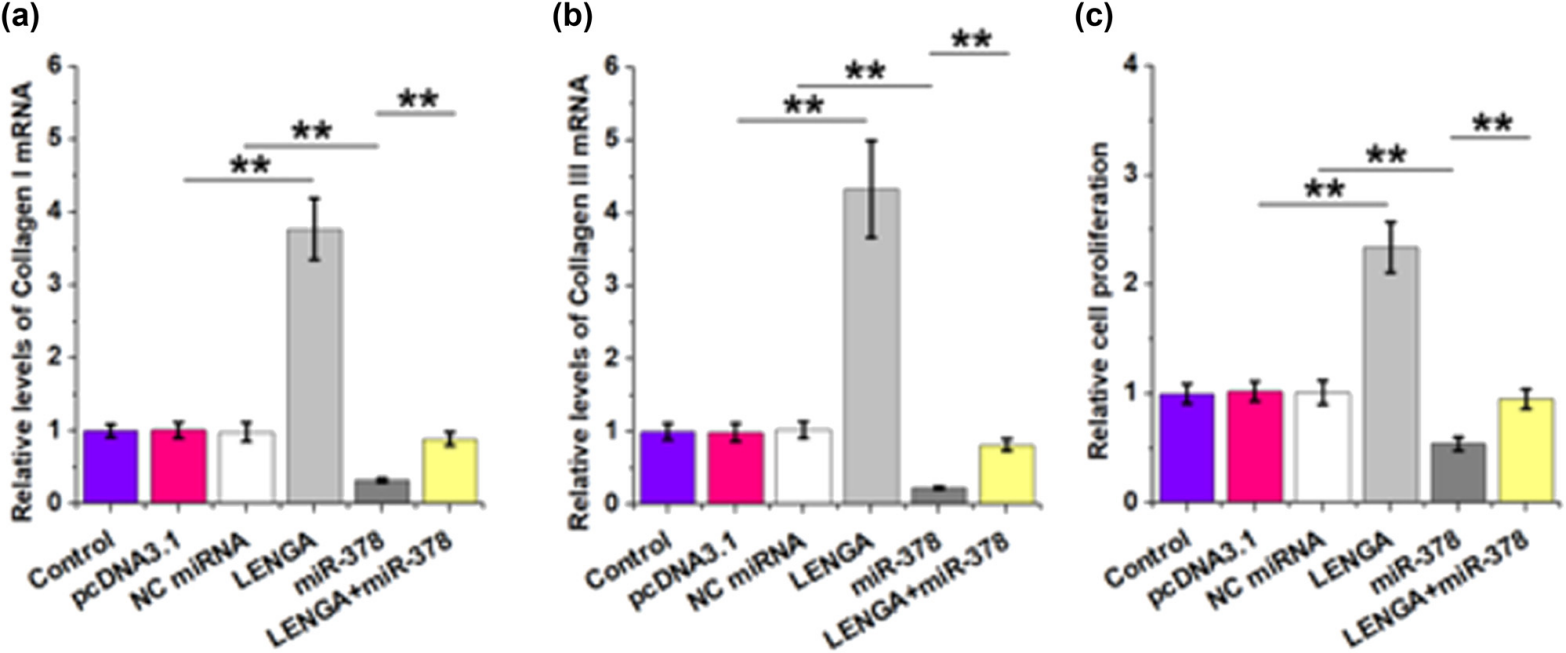
The role of LENGA in collagen production and cell proliferation was affected by miR-378. The roles of LENGA and miR-378 in regulating the expression of Collagen I and Collagen III mRNA (a and b) were analyzed by RT-qPCR as well as the proliferation of Ang-II-induced atrial fibroblasts were detected by BrdU assays (c), respectively. **p < 0.01.

The roles of LENGA and miR-378 in regulating the expression of Collagen I and Collagen III mRNA as well as the proliferation of Ang-II-induced atrial fibroblasts were analyzed by RT-qPCR and BrdU assays, respectively. LENGA promoted collagen production (Figure 4a, *p* < 0.01) and proliferation of Ang-II-induced atrial fibroblasts (Figure 4b, *p* < 0.01), while miR-378 played opposite roles. Moreover, LENGA suppressed the function of miR-378.

## 4 Discussion

The present study explored the involvement of LENGA and miR-378 in AF. We reported the altered expression of LENGA and miR-378 in AF, and their roles in regulating the expression of Collagen I and Collagen III and the proliferation of Ang-II-induced atrial fibroblasts. The potential interaction between these two ncRNAs has also been explored.

In a recent study, LENGA has been characterized as an lncRNA with tumor suppressor in gastric cancer, in which LENGA was downregulated and its overexpression interacts with BRD7/TP53 signaling to suppress tumor metastasis and growth [26]. To our best knowledge, the involvement of LENGA in other diseases has not been explored. The present study reported the increased expression of LENGA in AF. Moreover, LENGA shows a close correlation to Collagen I and Collagen III. Collagen turnover is involved in the perpetuation and generation of AF [27]. In the present study, overexpression of LENGA resulted in the increased expression of both Collagen I and Collagen III at mRNA level in atrial fibroblasts. Meanwhile, overexpression of LENGA also promoted the proliferation of Ang-II-induced atrial fibroblasts. Therefore, LENGA may affect collagen turnover and the proliferation of atrial fibroblasts to induce AF.

miRNAs have been reported to regulate many cell biological functions. In respect to AF, many miRNAs including miR-21, miR-29, miR-106b-25, miR-126, miR-409-3p, and miR-432 were reported to be involved in the pathogenesis of AF [18]. Moreover, miR-378 is known to inhibit myocardial fibrosis by a paracrine mechanism in mouse model [25]. However, its involvement in AF is unknown. Hence, in this study, miR-378 was found to be downregulated in patients with AF, and its overexpression suppressed the expression of both Collagen I and Collagen III in atrial fibroblasts. In addition, miR-378 also suppress the proliferation of Ang-II-induced atrial fibroblasts. Our study indicated the protective role of miR-378 in patients with AF.

Interestingly, LENGA was found to direct interact with miR-378, while they are not closely correlated with each other. Moreover, overexpression experiments showed that miR-378 and LENGA failed to affect the expression of each other. Interestingly, LENGA suppressed the role of miR-378 in the proliferation of Ang-II-induced atrial fibroblasts and the production of Collagen I and Collagen III mRNA in atrial fibroblasts. Therefore, LENGA may serve as an endogenous competing RNA for miR-378 to suppress its protective role in AF.

In summary, LENGA is overexpressed in AF and miR-378 is downregulated in AF. LENGA sponges miR-378 to promote the proliferation of Collagen I and Collagen III and increased the proliferation of Ang-II-induced atrial fibroblasts, thereby promoting the development of AF. LENGA and miR-378 are dysregulated in AF patients, thus leading to the conclusion that they can be used as potential biomarkers or therapeutic targets of AF.
